# The CRISPR/Cas9 system efficiently reverts the tumorigenic ability of *BCR/ABL in vitro* and in a xenograft model of chronic myeloid leukemia

**DOI:** 10.18632/oncotarget.15215

**Published:** 2017-02-09

**Authors:** Ignacio García-Tuñón, María Hernández-Sánchez, José Luis Ordoñez, Veronica Alonso-Pérez, Miguel Álamo-Quijada, Rocio Benito, Carmen Guerrero, Jesús María Hernández-Rivas, Manuel Sánchez-Martín

**Affiliations:** ^1^ Unidad de Diagnóstico Molecular y Celular del Cáncer, Centro de Investigación del Cáncer-IBMCC (USAL-CSIC), Salamanca, Spain; ^2^ IBSAL, Instituto de Investigación Biomédica de Salamanca, Salamanca, Spain; ^3^ Servicio de Hematología, Hospital Universitario de Salamanca, Salamanca, Spain; ^4^ Servicio de Transgénesis, Nucleus, Universidad de Salamanca, Salamanca, Spain; ^5^ Instituto Biología Molecular y Celular del Cáncer (USAL/CSIC), Salamanca, Spain; ^6^ Departamento de Medicina, Universidad de Salamanca, Salamanca, Spain

**Keywords:** CRISPR/Cas9, genome edition, BCR/ABL, leukemia

## Abstract

CRISPR/Cas9 technology was used to abrogate p210 oncoprotein expression in the Boff-p210 cell line, a pro-B line derived from interlukin-3-dependent Baf/3, that shows IL-3-independence arising from the constitutive expression of BCR-ABL p210. Using this approach, pools of Boff-p210-edited cells and single edited cell-derived clones were obtained and functionally studied *in vitro*. The loss of p210 expression in Boff-p210 cells resulted in the loss of ability to grow in the absence of IL-3, as the Baf/3 parental line, showing significantly increased apoptosis levels. Notably, in a single edited cell-derived clone carrying a frame-shift mutation that prevents p210 oncoprotein expression, the effects were even more drastic, resulting in cell death. These edited cells were injected subcutaneously in immunosuppressed mice and tumor growth was followed for three weeks. *BCR/ABL*-edited cells developed smaller tumors than those originating from unedited Boff-p210 parental cells. Interestingly, the single edited cell-derived clone was unable to develop tumors, similar to what is observed with the parental Baf/3 cell line.

CRISPR/Cas9 genomic editing technology allows the ablation of the *BCR/ABL* fusion gene, causing an absence of oncoprotein expression, and blocking its tumorigenic effects *in vitro* and in the *in vivo* xenograft model of CML. The future application of this approach in *in vivo* models of CML will allow us to more accurately assess the value of CRISPR/Cas9 technology as a new therapeutic tool that overcomes resistance to the usual treatments for CML patients.

## INTRODUCTION

Fusion proteins resulting from chromosomal rearrangements are known to drive the pathogenesis of a variety of hematological neoplasms such as chronic myeloid leukemia (CML). CML is a malignant myeloproliferative disorder driven by hematopoietic stem cells that acquire a reciprocal translocation between the long arms of chromosomes 9 and 22: t(9;22)(q34;q11) [1–3]. This translocation generates the BCR-ABL oncogenic fusion protein, which harbors constitutive tyrosine kinase activity. The characteristic expression of this oncoprotein transforms the hematopoietic progenitor cells by activating downstream signaling proteins that increase cell survival and proliferation [4].

CML treatments are based predominantly on the use of tyrosine kinase inhibitors (TKI), especially Imatinib mesylate, which have been highly effective at increasing the life expectancy of CML patients in recent decades. However, CML eradication is still hindered by the emergence of TKI resistant cells [5–7]. The mechanisms of Imatinib resistance can be BCR-ABL-dependent (gene amplification or point mutations) or BCR-ABL-independent [8]. A definitive and effective therapeutic strategy is therefore still needed for these patients [9].

Recently, Clustered Regularly Interspaced Short Palindromic Repeats (CRISPR)/Cas9 technology has initiated a new era of genome editing by overcoming the limitations of earlier methods [10, 11] that are inefficient, time-consuming and labor-intensive processes, hindering their general application [12, 13]. The CRISPR/Cas9 system has been widely used to generate genetic mutations in human cells [14–18] and to correct mutated genes [19–22]. The CRISPR/Cas9 system has created new possibilities for modeling diseases *in vitro* and *in vivo*, but few studies of CRISPR/Cas9 have explored the possibility of editing or correcting fusion genes resulting from chromosomal translocations [23–25].

*BCR/ABL* fusion, which is responsible for CML pathogenesis, is an ideal target for CRISPR-Cas9-mediated gene therapy. Moreover, the CRISPR/Cas9 system has been overwhelmingly adopted as a powerful tool for creating *in vivo* and *in vitro* models and new therapeutic opportunities for treating human diseases, including hematological conditions [26]. Thus, the present study shows a CRISPR-Cas9 application for truncating the specific BCR-ABL fusion (p210) at the genetic level in a cellular model. In addition, functional studies were performed to assess the effects of p210 on cell cycle and apoptosis. *In vivo* experiments were carried out to determine whether its tumorigenic capacity was also blocked by the use of mouse xenografts. To our knowledge, this is the first time that? CRISPR/Cas9 genomic editing system has been used to modify the *BCR/ABL* fusion gene successfully preventing its possible oncogenic effects.

## RESULTS

### The CRISPR/CAS9 system efficiently and specifically disrupts the human oncogene *BCR/ABL* fusion in an oncogene-dependent cell line

Boff-p210 is an oncogene-dependent cell line in which the expression of the human *BCR/ABL* oncogenic fusion confers the ability to survive and proliferate in the absence of IL-3 [27]. Cell cycle analysis of Boff-p210 cultured in the absence of IL-3 confirmed this capacity, in contrast to the Baf/3 un-manipulated parental cell line, which needs IL-3 to survive and proliferate [28]. The Boff-p210 genome was edited using CRISPR-Cas9 technology to truncate the *BCR/ABL* oncogene and inactivate its malignant potential.

**Figure 1:**
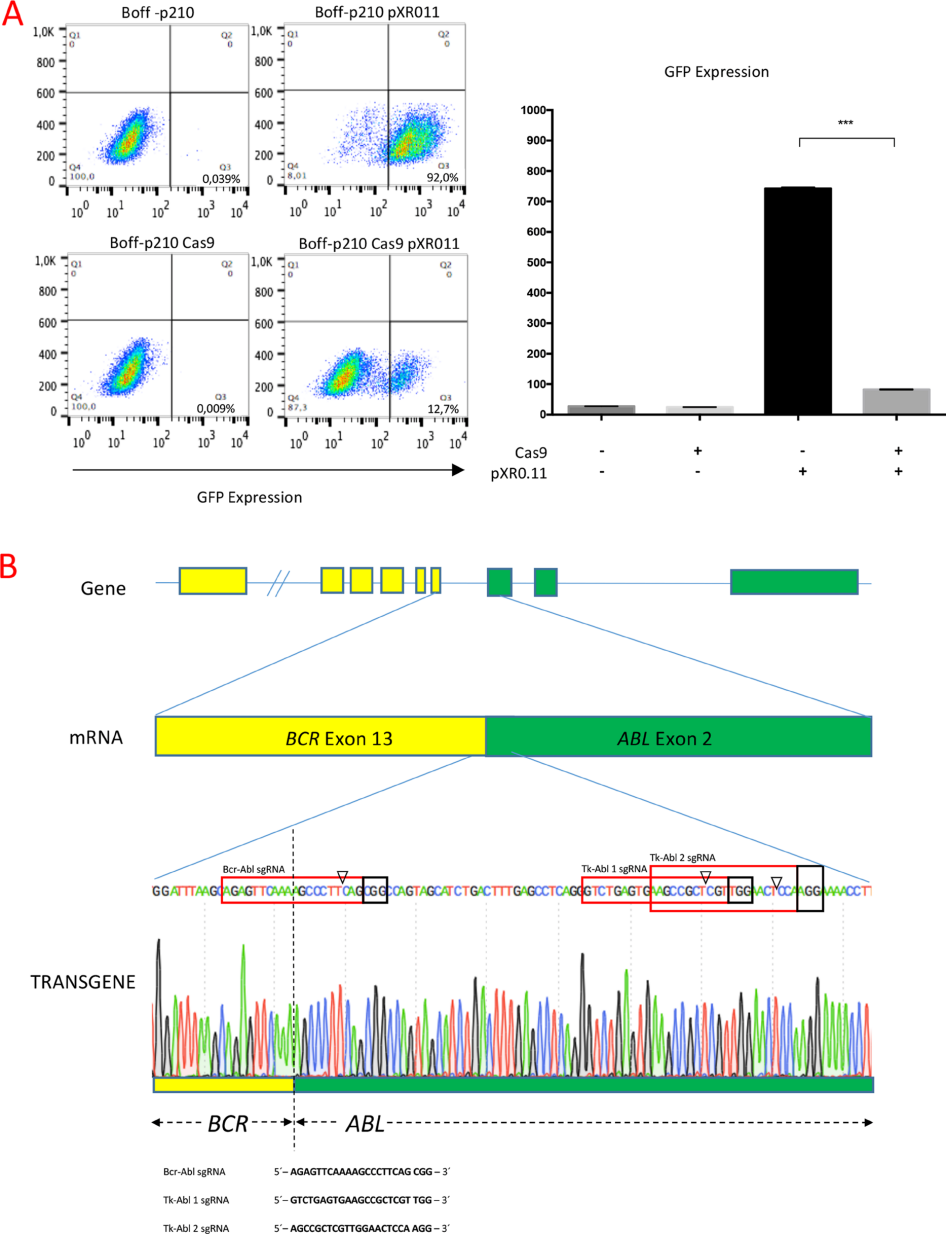
(**A**) Generation of the Boff-p210 Cas9 cell line. (Left panel) FACS plots showing the lower frequency of GFP-positive cells in Cas9-expressing cells compared with parental cells, both transduced with pXPR011. (Right panel) Quantification of GFP expression (mean ± SD). (**B**) Schematic representation of BCR/ABL fusion transgene and the sequences of sgRNAs for editing. Schematic representation of the *BCR/ABL* fusion transgene, and the sequences of three sgRNAs designed to edit *BCR/ABL*. One of them, Bcr-Abl sgRNA (highlighted in the red box) had 10 bp complementary to the *BCR* sequence and 10 bp complementary to the *ABL* gene, providing high specificity with the fusion gene sequence. Tk-Abl 1 and Tk-Abl 2 sgRNA were complementary to the *ABL* sequence. The arrowhead indicates the expected Cas9 cleavage site. PAM (highlighted in the black box) is the protospacer-adjacent motif required for Cas9 nuclease activity.

To assess human gene editing using CRISPR/Cas9 technology in a p210 oncogene-dependent cell line, Boff-p210 cells were transduced with a lentivirus expressing a constitutive Cas9, thereby establishing the Boff-p210 Cas9 cell line. Cas9 nuclease activity was then assessed by transducing Boff-p210 Cas9 and its parental cell line with a plasmid encoding both GFP and the sgRNA against GFP [29]. After ten days, FACS analysis showed, upon transduction with this vector, an ~80% reduction in the frequency of GFP-positive cells in the active Cas9-expressing cell line compared with Boff-p210 parental cells (Figure 1A), indicating an efficient expression of active Cas9 nuclease in Boff-p210 Cas9 cells.

Three custom-designed single guide RNAs (sgRNAs) were used to genetically inactivate the *BCR/ABL* oncogene. These specific sgRNAs direct Cas9 to the *BCR/ABL* fusion sequence (Bcr-Abl sgRNA) or to the Abelson tyrosine kinase sequence (Tk-Abl 1 sgRNA and Tk-Abl 2 sgRNA), 40 nucleotides downstream of the fusion point (Figure 1B). Three individual lentiviral infection assays were performed with each sgRNA to generate three different Boff-p210 clones with the edited *BCR/ABL* oncogene, establishing three edited pools of Boff-p210 Cas9 cells.

**Figure 2:**
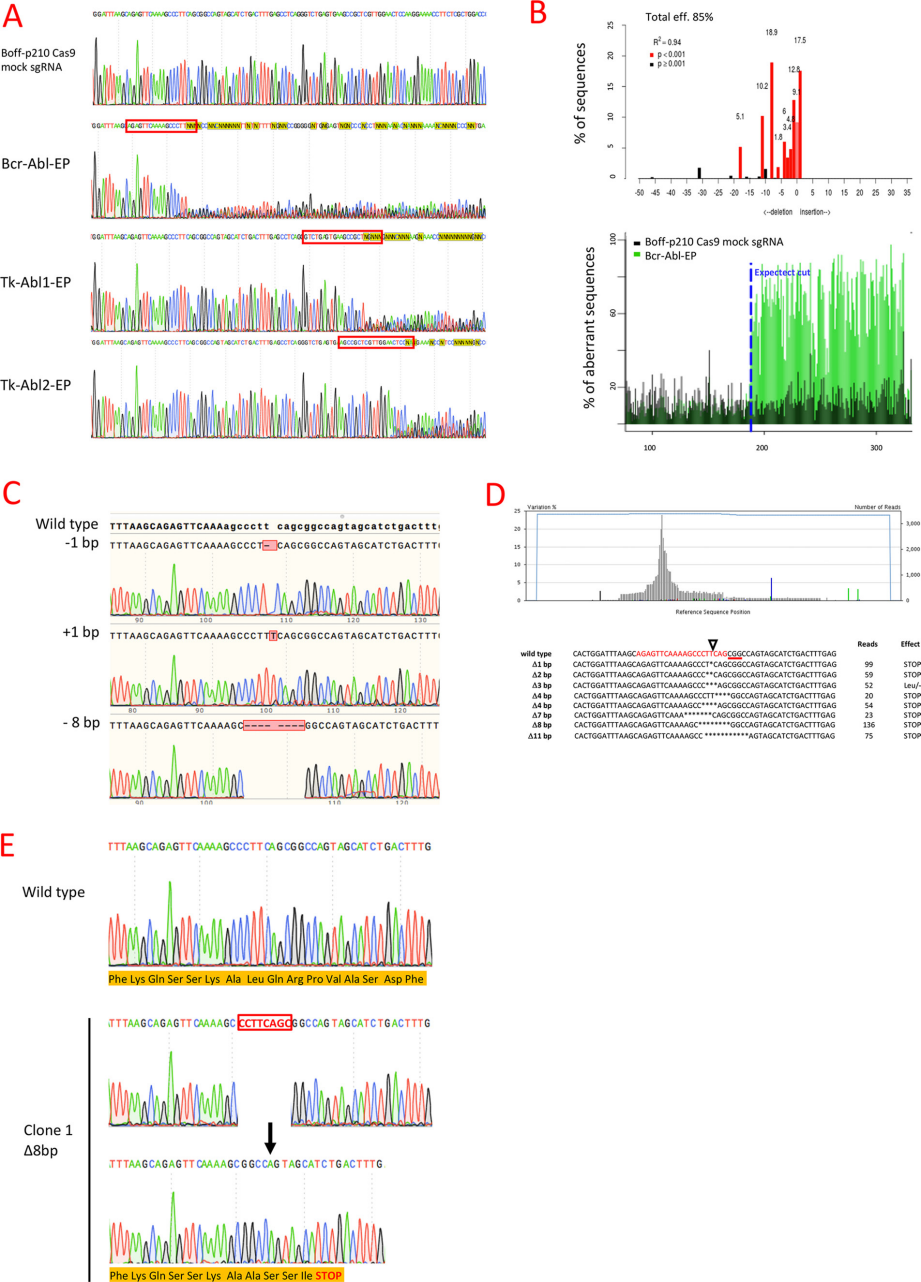
Genome editing of BCR/ABL in the Boff-P210 cell line (**A**) Sanger sequencing of the *BCR/ABL* fusion region in Boff-p210 cells. The Boff-p210 cells expressing mock sgRNA, used as a control, had a wild type sequence, while cells expressing Bcr-Abl sgRNA (Bcr-Abl-EP), Tk-Abl1 sgRNA (Tk-Abl1-EP) and Tk-Abl2 sgRNA (Tk-Abl1-EP) showed a mixture of sequences around the expected Cas9 cleavage point. (**B**) TIDE decomposition algorithm analysis of the edited sequence in Bcr-Abl-EP cells, showing high editing efficiency at the expected cleavage point. The lower panel illustrates the aberrant sequence signal in Boff-p210 cells (black) and Boff-p210-edited cells (green) and the expected cleavage site (vertical dotted line). (**C**) DNA sequences of DNA post-PCR cloning colonies of Boff-p210-edited cell DNA. We compare three induced mutations with the original sequence. (**D**) Next-generation sequencing of the transgene target region. The upper panel shows the percentages of each base pair in the sequenced region. The lower panel shows commonly sequenced variants for CRISPR-targeted *Bcr/Abl* transgene in Boff-p210 cells. The sgRNA-target site is displayed in red, the PAM sequence as text underlined in red, and the cleavage site is indicated by a triangle. The number of reads per variant and the predicted effect of the mutation are shown. (**E**) In silico analysis of single cell-derived clone. Clone 1 showed a 8 bp deletion resulting in a stop codon and in a premature end of translation. It was selected to establish the Bcr-Abl-SC cell line.

Sanger sequencing showed the presence of indel mutations at the expected locations in all the CRISPR-Cas9 assays with each p210 sgRNA, while no changes were observed with mock sgRNA (Figure 2A). Tracking of Indels by Decomposition (TIDE) analysis identified BCR-ABL sgRNA as the most efficient sgRNA of those tested, with 85% of the Boff-p210 Cas9 cell pool edited (Bcr-Abl-EP hereafter) (Figure 2B, Table 1). Likewise, the algorithm predicted different patterns of genome repair, mainly deletions in the 11 bases adjacent to the cleavage point. The most frequently predicted mutations were an 8-bp deletion (18.5%), a 1-bp insertion (17.5%), an 11-bp deletion (10.2%) and a 1-bp deletion (9.1%) (Figure 2B, Table 1).

**Table 1:** TIDE algorithm predicted indels induced by each sgRNA

Guide	Edition efficiency	TIDE Predicted Indels
Bcr-Abl sgRNA	85%	+1 bp (17.5%), −1 bp (9.1%), −2 bp (4.8%) −3 bp (3.4%), −4 bp (6%), −6 bp (1.8%), −8 bp (18.9%), −11 bp (10.2%), −18 bp (5.1%)
TK-ABL 1 sgRNA	54,6%	+1 bp (14.9%), −1 bp (8%) −2 bp (5.2%), −10 bp (17.6%)
TK-ABL 2 sgRNA	68,8%	+1 bp (30.8%), −1 bp (5.9%), −2 bp (4.8%), −4 bp (15.2%), −14 bp (5.1%)
Mock sgRNA	0%	

Similar indels were obtained with the TK-Ab l 1 and TK-Abl 2 sgRNAs, although the overall predicted editing efficiencies (54.6% and 68.8%, respectively) were lower than that of Bcr-Abl sgRNA (Table 1). Thus, Bcr-Abl sgRNA was selected to confirm the indel mutations generated by the CRISPR/Cas9 system. Target DNA from the Bcr-Abl-EP cells was subcloned by PCR. Colonies were sequenced by the Sanger method to confirm that the mutations carried by the clones corresponded to the alterations predicted by the TIDE algorithm: 1-bp insertion, 1-bp or 8-bp deletions (Figure 2C). These findings suggest that these mutations are frequently generated when the cell repairs the Cas9-induced cleavage. Moreover, next-generation sequencing (NGS) was performed to sequence the target region of the Bcr-Abl-EP cells (coverage = 3386X). Several indels were detected around the expected Cas9 cleavage point (Figure 2D). The most frequent indels (> 5%) were frame-shift mutations producing a stop codon, except for the TTC-3bp-deletion (52 reads), which caused the loss of a leucine, but did not alter the downstream reading frame (Figure 2D). Therefore, most of the edited Bcr-Abl-EP cells showed mutations that produce truncated p210 oncoprotein (Figure 2D).

It is of note that no altered sequences were found in any of the top five potential off-target genes of Bcr-Abl sgRNA (Table 2), suggesting that the activity of the CRISPR/Cas9 system designed in our experimental procedures is highly specific.

**Table 2:** Analysis of the genomic potential off-targets of Bcr-Abl sgRNA

Gene	Annotation	Target	Forward	Reverse
**Rcor1**	NM_198023	GGAAATCACAAGCCCTTCAGCAG	ATGGCAGCTCTCCCAATCAACA	TACAAGCACCAGGTACAATGA
**Epg5**	NM_001195633	AGTGTTGAATAACCCTTCAGGGG	CAGTAGCAGTGTTGCCCTCCA	CACACCTTCTCTTAGTCTATGG
**Gimap7**	NM_146167	AGAACTCAAAGGCCCTGCAGGAG	CTAAGCAACATGTGGACCACT	GGAGCTGAAGGGAGTTAGGG
**Zfp457**	NM_001003666	AGTGTACAAAAGCTCTTCAGTGG	GCGACTAAGATGGTTTCAATAG	GCACAGCACCTCAAGGTCAG
**Alg10b**	NM_001033441	AGAGCCCACAACCCCTTCAGCGG	AGTGCTCCTAAGGGGCTTAGT	GAAGTAAGCGGCATTGCTTATAG

### Establishment of a single cell derived-cell line carriying a specific CRISPR/CAS9-mediated mutation in the *BCR/ABL* oncogene

To generate a clone of Boff-p210 cells with truncated BCR-ABL oncoprotein, single cells from the Bcr-Abl-EP cell line, were sorted by FACS. 41 single-cell clones were analyzed by Sanger sequencing, which revealed that most of them (32/41) had an edited sequence (Supplementary Table 1). Four of these 32 clones, showing indel mutations that generated a premature stop codon near the Cas9 cleavage point, were choose to functional analysis (Supplementary Figure 2A). One of them (Clone 1), which carried an 8-bp deletion at the expected cleavage point, was selected to establish the cell line known as Bcr-Abl-SC (single edited cell-derived Boff-p210-Cas9), whose frame-shift mutation was predicted by the TIDE algorithm and confirmed by both PCR-subcloning and NGS (Figure 2E). In silico analysis of the mutation in clone 1 resulted in the generation of a stop codon downstream of the Cas9 cleavage point, leading to the alteration of the open reading frame and the subsequently premature end of translation (Figure 2E).

### Obliteration of the *BCR/ABL* oncogene by CRISPR-CAS9 in the BOFF-P210 cell line leads to an absence of the oncogenic protein

**Figure 3:**
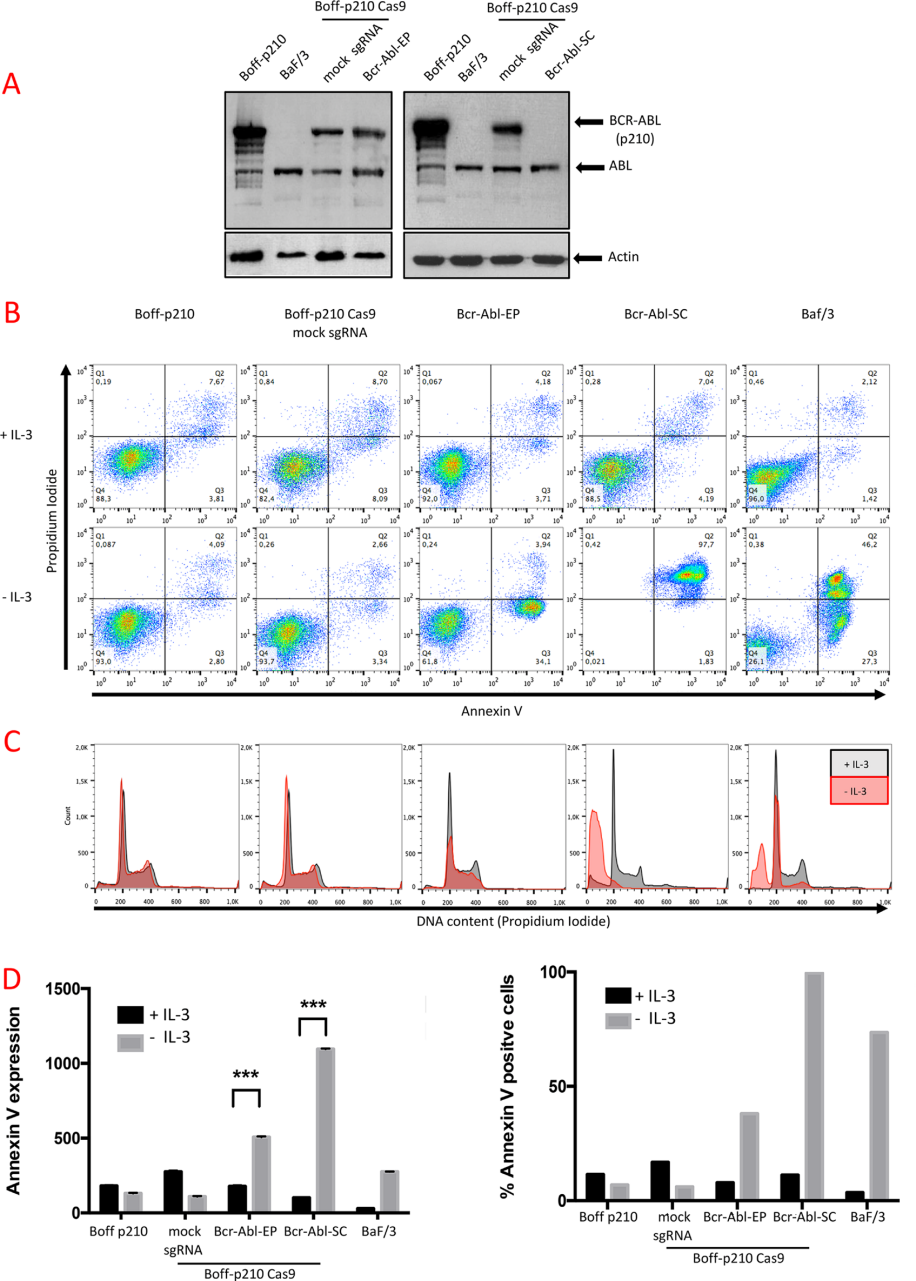
Functional analysis of Boff-p210-edited cells (**A**) Western blot analysis of BCR-ABL expression. Abl expression (140 kDa) was observed in all cells. A 210 kDa band, corresponding BCR-ABL expression was observed in unedited Boff-p210 cells (parental and sgRNA mock controls) and in Bcr-Abl-EP cells. In contrast, Bcr-Abl-SC and Baf/3 cells showed no expression of BCR-ABL. (**B**) Annexin V/propidium iodide labeling of Boff-p210 cells after four days in culture in the presence or absence of IL-3. Unedited and mock sgRNA-expressing cells showed IL-3-independent growth. Baf/3 as Bcr-Abl-EP cells showed greater annexin V labeling after IL-3 withdrawal. This effect was stronger in cell lines derived from a single-edited cell. (**C**) Flow cytometric analysis of Bcr-Abl-EP cells. No changes were observed in the cell cycle phases of Boff-p210 or the mock cells, grown with or without IL-3. High frequencies of cells in the subG0 phase were found in the single edit derived-cells cultured without IL-3, as in Baf/3 cells. (**D**) Quantification of annexin V labeling (Left graph, represented as mean ± SEM; ***p < 0.001) and percentage of Boff-p210-positive cells (right graph).

To confirm that the CRISPR-Cas9 system efficiently truncates *BCR/ABL* oncogene, the presence of the BCR-ABL oncoprotein was tested by western blot analysis. A single band, corresponding to the ABL protein (140 kDa), was detected in Baf/3 (Figure 3A), while Boff-p210 also showed a 210-kDa band corresponding to a BCR-ABL fusion. No changes were observed in either *BCR-ABL* or *ABL* expression in pools of edited cells (Figure 3A). Similar results were obtained for the TK-Abl sgRNAs cell lines (Supplementary Figure 1A). In contrast, BCR-ABL protein expression was undetectable in the Bcr-Abl-SC cells (Figure 3A, Supplementary Figure 2B).

### *In vitro* ablation of *BCR/ABL* by the CRISPR/CAS9 system in tumorigenic p210 cells induces cell cycle arrest and impairs survival

To study the consequences of *in vitro* obliteration of the *BCR/ABL* oncogene by the CRISPR-Cas9 system, the processes of cell-cycle progression and apoptosis were analyzed in Boff-p210-edited cells in the presence or absence of IL-3 for 4 days.

Annexin V and PI labeling were used to measure apoptosis in Boff-p210 cells by flow cytometry after 4 days in culture in the presence or absence of IL-3 (Figure 3B). We simultaneously analyzed the distribution of the cells in the different cell cycle phases by permeabilization followed by PI staining (Figure 3C). Non-tumorigenic Baf/3 cells rapidly died after 4 days in IL-3-withdrawn medium (Figure 3A). After 4 days of IL-3 deprivation, 73.3% of the Baf/f3 cells showed annexin V staining (Figure 3B, 3D), and there was clear evidence of oligonucleosomal DNA degradation (subG0 DNA content), characteristic of apoptosis (Figure 3C). In contrast, only 10–20% of the Boff-p210 or Boff-p210 Cas9 mock sgRNA cells, growing with or without IL-3, were positive for annexin V staining (Figure 3B, 3D). Similarly, no differences in the distribution of the cell cycle phases were observed in Boff-p210 and mock cells in the presence or absence of IL-3 (Figure 3C), with no evidence of cells in sub-G0. However, withdrawal of IL-3 in Bcr-Abl-EP cell cultures resulted in increased annexin V staining (7.89% in medium with IL-3; 38.04% in the absence of IL3), indicating that edited cells are IL3-dependent (Figure 3B, 3D). Accordingly, we observed a decrease in the G1, S and G2/M phases of the cell cycle in Bcr-Abl-EP cells in the absence of IL-3, while the presence of IL-3 maintained the cell viability of these cells. Boff-p210 and non-edited Boff-p210 cells (mock sgRNA assay) yielded similar results in the distribution of the cell cycle phases with or without IL3-conditioned medium (Figure 3C). A similar effect was observed in TK-ABL1-EP and TK-ABL2-EP cells in the absence of IL-3 (Supplementary Figure 1B).

To confirm that the loss of ability to grow in the absence of IL-3 observed in the pool of edited cells was due solely to the disruption of the *BCR/ABL* transgene, we assessed the effect of IL-3 withdrawal in an edited cell line derived from a single cell (Bcr-Abl-SC cells). Like control mock and Bcr-Abl-EP cells, Bcr-Abl-SC cells showed low expression of annexin V (11.23% of cells) and high viability in the presence of IL-3 (Figure 3B, 3C, 3D). However, after 4 days in IL-3-deprived culture, most cells (97.7%) showed a high level of annexin V staining (Figure 3B, 3D), accompanied by fragmented DNA (Figure 3C). The dramatic effect observed in the Bcr-Abl-SC cell line, similar to that of Baf/3 cells, suggests that there is a complete lack of BCR-ABL functional oncoprotein. These results were confirmed in three clones derived from a single-edited cell carrying a truncated *BCR/ABL* sequence in which IL-3 withdrawal produced a similar drastic effect (Supplementary Figure 2C).

### CRISPR-CAS9 impairs the tumorigenic capacity of edited Boff-p210 selected cells

**Figure 4:**
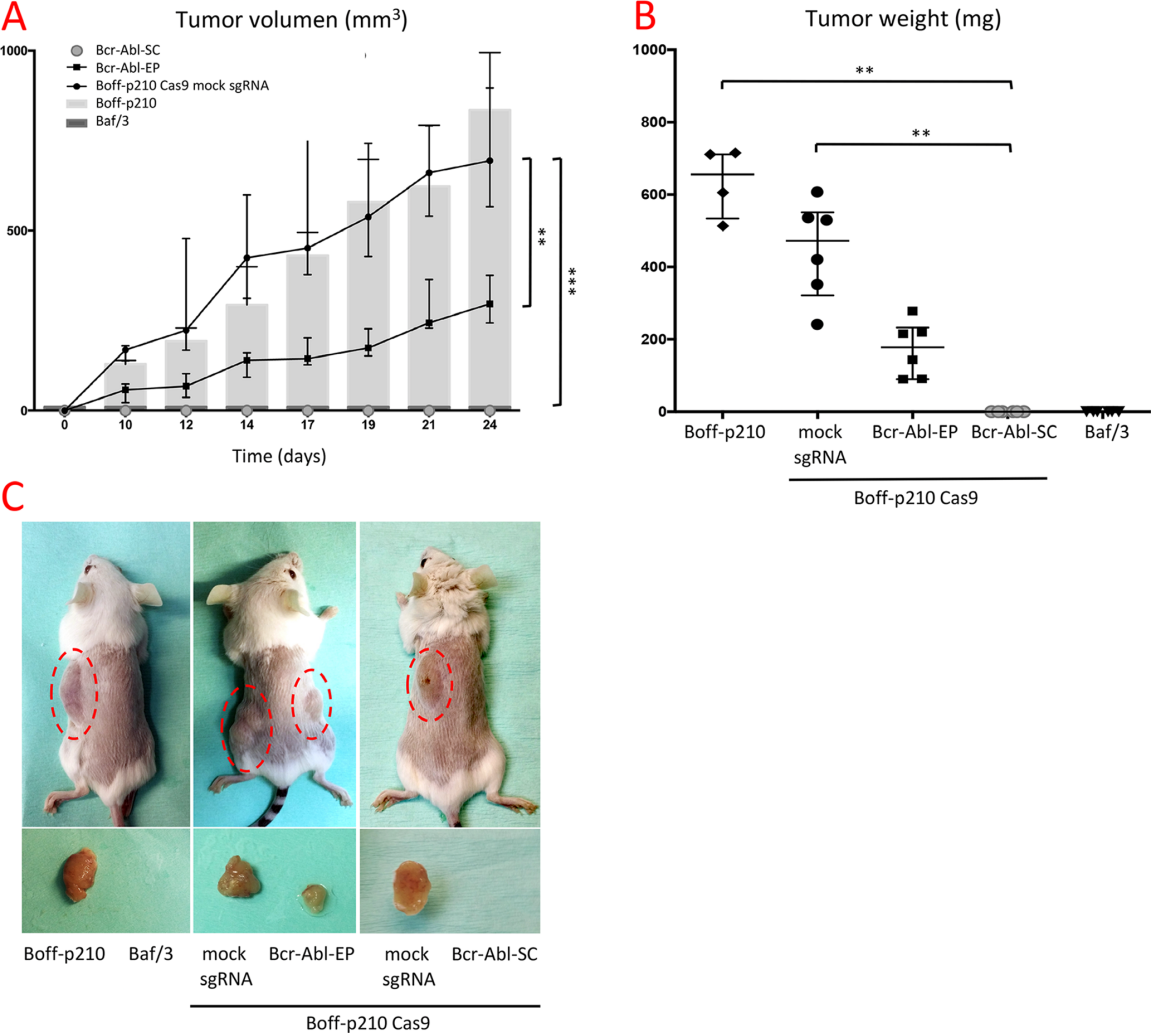
*In vivo* effects of CRISPR-mediated editing of the BCR/ABL oncogene (**A**) Tumor growth (mm3) over the 24 days following subcutaneous cell injection. Similar tumor growth was observed in Boff-p210 (grey bars) and Boff-p210 Cas9 mock sgRNA (black dots line) injected cells. Tumors formed by Bcr-Abl-EP cells (black squares line) were half the size of those induced previously. Tumor growth was observed when the single edited cell-derived cells were injected (grey dotted line), as well Baf/3 cells (dark grey bars). The plot shows medians and ranges; ***p < 0.001). (**B**) After 24 days, mice were sacrificed and their tumor mass measured. The final tumor mass was reduced by half in the case of the edited pool cells (black squares), relative to controls (black dots). Bcr-Abl-SC (grey dots) and Baf/3 (black triangles) cells were unable to form a subcutaneous tumor. The plot shows medians and ranges; **p < 0.05). (**C**) External appearance of mice and developed tumors 24 days after subcutaneous cell injection.

In order to determine the effects of Bcr/Abl oncogene disruption *in vivo*, the flanks of CB17C SCID mice were subcutaneously injected with Baf/3, Boff-p210, Bcr-Abl-EP cells, and Boff-p210 Cas9 mock sgRNA (Boff-p210 mock) cells. Mice injected with Boff-p210 cells and Boff-p210 mock cells, developed similar detectable tumors (mean mass: 663 mg and 445 mg respectively). In contrast, mice injected with Bcr-Abl-EP cells gave rise to significantly smaller (around 60%) subcutaneous tumors than those produced by the non-edited cells (mean mass: 172 mg; Figure 4A and 4B). As expected, Baf/3 cells showed no tumor growth in this period.

Bcr-Abl-SC cells carrying an 8-bp deletion (clone 1, Supplementary Figure 2) were selected to test their tumorigenic capacity by injection into the flank of CB17C SCID mice. Non-edited Boff-p210 cells and Baf/3 cells were also injected as controls. 24 days post-injection, tumor growth was clearly detected in mice injected with non-edited Boff-p210 cells. Conversely, no tumor growth was observed in mice injected with cells derived from the single-edited cell line, or with Baf/3 cells (Figure 4A).

## DISCUSSION

In this study, we have explored the ability use of the CRISPR/Cas9 technology to obliterate BCR-ABL fusion in order to determine its impact on the leukemic processes in *in vitro* and in xenograft models of CML. The CRISPR/Cas9 system has recently emerged as a powerful genome editing tool [14, 30]. Cas9 nuclease from *Streptococcus pyogenes* can be guided by means of simple base-pair complementarity between the first 20 nucleotides of an engineered sgRNA and a target genomic DNA sequence of interest [31, 32]. Recently, this technology has been used for gene correction of point mutations in cells [20, 21, 33]. However, to our knowledge, this is the first study in which CRISPR/Cas9 has been used to prevent the tumorigenic ability of an oncogenic fusion, such *BCR/ABL*, by inducing frame-shift mutations.

It is well known that the crucial genetic event in CML development is the generation of a t(9;22)(q34;q11) reciprocal chromosomal translocation [34], resulting mainly in the generation of the BCR-ABL (p210) oncoprotein with a constitutive kinase activity [35–37]. Herein, the tumorigenic Boff-p210 cell line is used as a cellular model, which is particularly suitable for BCR-ABL assays. This is an interleukin 3- (IL-3) independent cell line derived from the Baf/3 hematopoietic cell line [38]. Boff-p210 expresses the BCR-ABLp210 oncogene as a tetracycline-regulated transgene and can be genetically modified, expanded and then re-administered into immunocompromised mice. In the absence of tetracycline or doxycycline, *BCR/ABL* expression is constitutive, and confers on Boff-210 cells the ability to survive and proliferate in the absence of IL-3 [39].

A Boff-p210 cell line expressing constitutively active Cas9 was generated to genetically inactivate human *BCR/ABL* oncogenes, thereby to avoid its pathological effect on the Boff-p210 cell line. Three specific sgRNAs against the *BCR/ABL* fusion gene were designed as RNA guides for Cas9. One of them drives Cas9 nuclease against the *BCR/ABL* junction sequence and the other two drive it against the *ABL* tyrosine kinase sequence. The CRISPR-Cas9 system was highly effective at inducing indels in their target sequences with all the sgRNAs used. Notably, Bcr-Abl sgRNA was the most efficient, involving up to 80% of the edited cells.

Since CRISPR/Cas9 nuclease can induce off-target cleavages, due to unspecific recognition of non-target sequences [40], the integrity of the main off-target sequences was tested. It is worth pointing out that no off-target sequences were observed in any of the edited pools of cells. These findings suggest that the CRISPR/Cas9 system is highly specific, as has been demonstrated by earlier whole-genome sequencing studies [41–43].

The effects of the constitutive *BCR/ABL* activity are pleiotropic and promote leukemogenesis by acquisition of tumor abilities. These abilities include the increase of cell survival [4], the avoidance of apoptosis [39, 44, 45] and the promotion of genomic instability by downregulation of DNA-repair mechanisms [46, 47]. In this cellular model, the expression of BCR-ABL oncoprotein induces IL-3-independent proliferation and survival, while Baf/3 cells require IL-3 to survive and proliferate (Figure 3B–3D). To study the oncogene-disruption effect in this cellular model, CRISPR-Cas9 edition assays were performed. The CRISPR-Cas9 system efficiently induced different mutations at the expected cleavage expected point, giving rise to a cellular pool with several altered *BCR/ABL* sequences. A significant increase in cell death was observed in Bcr-Abl-EP cells in the absence of IL- 3, compared with Boff-p210 parental cells or mock Boff-p210 cells. However, a massive cell death was not observed in Bcr-Abl-EP cells, indicating the presence of edited cells with survival capacity in the absence of IL-3. This finding was corroborated by the fact that Bcr-Abl-EP did not show any significant variations in the level of p210 expression as shown by western blot analysis. As confirmed by NGS, the CRISPR-Cas9 system induced several different indel mutations in our edited cell lines. Some of them were frame-shift mutations that resulted in truncated proteins, whereas others generated a gain/loss of amino acids that gave rise to a functional protein. These functional repaired p210 proteins could protect cells from apoptosis induced by IL-3 withdrawal, promoting the cell survival of several clones. This result is consistent with previous reports about the antiapoptotic effect of the *BCR/ABL* [44, 45]. To avoid this drawback, a single cell with a specific edited sequence of *BCR/ABL* was isolated from the edited pool. In particular, a specific single-cell clone with an 8-bp deletion at the cleavage point was selected for further experiments. The selected cell clone had an edited p210 sequence, which gave rise to a premature translation end. In fact, no BCR-ABL oncoprotein expression was observed by western blot. Interestingly, this single edited cell-derived clone showed the highest level of apoptosis (more than 97% of cells) when it grew in the absence of IL-3, compared with that observed in cells that did not express p210.

To further test the tumorigenic capability of the BCR-ABL-edited cells, the xenograft model was generated by injecting these edited cells into mice. In all cases, the pool of Boff-p210-edited cells gave rise to a subcutaneous tumor 24 days post-injection. These findings indicate that cells with functional BCR-ABL oncoproteins maintain their tumorigenic capabilities. In contrast, cell clones derived from a single-edited cell were unable to induce tumors in all cases. This result demonstrates that CRISPR-Cas9 could efficiently prevent the tumorigenic potential of the p210 oncoprotein.

Despite major improvements in therapeutic strategies in patients with CML in recent decades, as a consequence of the use of tyrosine kinase inhibitors, eradication of CML remains a challenge because of the emergence of drug resistance. In the context of stem cell transplantation [48], the use of CRISPR-Cas9 technology could be a promising new therapeutic option for CML patients who have developed tyrosine-kinase resistance. Bone marrow leukemic stem cells could be edited by CRISPR-Cas9 technology to truncate BCRL/ABL fusions, and truncated cells specifically selected before infusion. However, the fact that of sgRNAs can tolerate certain mismatches and that Cas9 is integrated and constantly produced represent the key limit of this technology. The use of integrase-deficient lentivirus vectors or Cas9-guide RNA ribonucleoprotein (RNP) complexes into primary cells, could represent a safety advantage in human therapy [49–51]. Further studies with human primary cells should be performed to test this hypothesis.

Our study is the first step towards providing proof-of-principle for genome editing of the main genetic event in CML patients. In summary, this is the first report describing the use of CRISPR/Cas9 genome editing to completely truncate human BCR-ABL oncoprotein fusion in order to prevent its tumorigenic abilities in an *in vitro* as well as in an *in vivo* CML model.

## MATERIALS AND METHODS

### Cell lines and culture conditions

Boff-p210 is a murine interleukin 3 (IL3) -independent cell line derived from the hematopoietic cell line Baf/3 [38] that expresses *BCR/ABL* as a tetracycline-regulated transgene (*BCR/ABL* expression is constitutive in the absence of tetracycline or doxycycline) [52–54]. Boff-p210 was maintained in Dulbecco's Modified Eagle's Medium (DMEM) (Life Technologies) supplemented with 10% fetal bovine serum (FBS) and 1% of penicillin/streptomycin (Life Technologies) in 5% CO_2_ at 37°C. IL-3-dependent Baf/3 cells, used as a parental cell line, were grown in the same medium supplemented with 10% WEHI-3-conditioned medium as a source of IL-3.

HEK 293T cells were used for lentivirus production. They were maintained in RPMI 1640 (Life Technologies) supplemented with 10% FBS and 1% penicillin/streptomycin (Life Technologies).

### Lentiviral constructs, sgRNA design and sgRNA cloning

The constitutive Cas9 expression vector (plasmid LentiCas9-Blast, a gift from Feng Zhang, Addgene plasmid # 52962) [55] was used to generate the Boff-p210 cell line with stable Cas9 expression.

pLKO5.sgRNA.EFS.GFP (Addgene plasmid # 57822) [56] containing the coding sequence of GFP and a cloning site for sgRNA sequence was digested with Esp3I (BsmBI) (NEB). To clone the sgRNAs into the pLKO5 vector, two complementary oligos for each sgRNA were designed including two 4bp-overhang sequences (Table 3). The sgRNAs sequences were designed with the Broad Institute CRISPR designs software (http://www.broadinstitute.org/rnai/public/analysis-tools/sgrna-design). One sgRNA (Bcr-Abl sgRNA) was designed to specifically target the fusion junction sequence. To control for variability in targeting efficiency and potential off-targets effects, two sgRNAs were designed to target the *ABL* tyrosine kinase sequence (Tk-Abl1 sgRNA and Tk-Abl2 sgRNA) (Figure 1B). An sgRNA designed not to target the genome was used as a negative control (mock). The two complementary oligos were denatured at 95°C for 5 min, ramp cooled to 25°C over a period of 45 min to allow annealing, and finally ligated with the linearized pLKO5. Competent cells were transformed with 2 μl of the ligated plasmid, and single colonies were expanded before to plasmid extraction using a QIAprep Spin Maxiprep kit (Qiagen). The correct insertion of the sgRNA sequences was confirmed by using Sanger sequencing.

**Table 3:** Oligos designed for each sgRNA

	Forward	Reverse
**Bcr-Abl**	CACCGAGAGTTCAAAAGCCCTTCAG	AAACCTGAAGGGCTTTTGAACTCTC
**TK-Abl 1**	CACCGAGCCGCTCGTTGGAACTCCA	AAACTGGAGTTCCAACGAGCGGCTC
**TK-Abl 2**	CACCGGTCTGAGTGAAGCCGCTCGT	AAACACGAGCGGCTTCACTCAGACC
**mock**	CACCGACGGAGGCTAAGCGTCGCAA	AAACTTGCGACGCTTAGCCTCCGTC

Cas9 activity was tested using a previously reported system based on the pXPR-011 plasmid (a gift from John Doench and David Root; Addgene plasmid #59702) which delivers GFP and sgRNA targeting GFP [29].

### Lentiviral production and cell transduction

Lentiviral particles were produced by transient transfection of HEK 293T cells using Lipofectamine 2000^®^ (Life Technologies). Viral constructs were co-transfected with pMD2.G (Addgene plasmid 12259) and psPAX2 (Addgene plasmid 12260) (both kindly provided by Didier Trono, EPFL, Lausanne, Switzerland). Lentiviral particles were collected at 24 and 48 h, and, finally, were concentrated using Lenti-X concentrator^®^ (Clontech). Cells were infected in 24-well plate format, with each well containing 2 × 10^5^ cells, cultured in media supplemented with 4 μg/mL polybrene.

### Cas9 activity assay system

To obtain stable Boff-p210 cells expressing Cas9, lentiviral particles containing LentiCas9-Blast, plasmid-expressing Cas9 and blasticidine resistance (a gift from Feng Zhang, Addgene plasmid # 52962) [55], were transduced into Boff-p210 cells and selected by blasticidine (10 μg/μl), over ten days.

Cas9 activity was assessed using the Cas9-activity vector as previously reported [29]. The Cas9-expressing Boff-p210 cell line and its corresponding parental Boff-p210 cell line were transduced with plasmid pXPR-011, encoding both GFP and sgRNA against GFP (a gift from John Doench and David Root; Addgene plasmid # 59702) [29]. 48 h post-infection, 2 μg/mL puromycin was added and cells were selected for three days. GFP-positive cells from both cell lines were analyzed by BD FACScalibur ten days post-infection.

### Sequencing of sgRNA targets sites

Genomic DNA was extracted using the QIAamp DNA Micro Kit (Qiagen) following the manufacturer's protocol. To amplify the region of the BCR-ABL fusion, PCR was performed using the following primers: forward 5′- TCGTGTGTGAAACTCCAGACTGTC – 3′ and reverse 5′- TTGGGCTTCACACCATTCCCC – 3′. PCR products were purified using a High Pure PCR Product Purification Kit (Roche) and were sequenced by the Sanger method using each forward and reverse PCR primers.

The editing efficiency of the sgRNAs and the potential induced mutations were assessed using Tracking of Indels by Decomposition (TIDE) software (https://tide-calculator.nki.nl; Netherlands Cancer Institute), which only required two Sanger sequencing runs from wild-type cells and mutated cells.

To identify specifically the different generated mutations, total DNA of Cas9-edited cells were PCR-amplified, subcloned and transformed in bacteria. DNA from single clones were extracted with a QIAprep Spin Miniprep Kit (Qiagen) and sequenced by Sanger sequencing.

In parallel, Next Generation Sequencing (NGS) technology was employed with the same Sanger primers with the corresponding adapters added, to read each edited sequence individually. The amplicon libraries were sequenced on the GS Junior platform (454 Life Sciences, Roche, Branford, CT, USA) [57].

### Western blot

BCR-ABL protein expression was assessed by SDS-PAGE and western blotting using a mouse anti-ABL antibody (1:1000; SC-23, Santa Cruz). Horseradish peroxidase-conjugated α-mouse antibody (1:10000; NA931V, GE Healthcare) was used as a secondary antibody. Antibodies were detected using ECL^TM^ Western Blotting Detection Reagents (RPN2209, GE Healthcare).

### Flow cytometry analysis and cell sorting for Cas9-mediated editing and for isolating single edited cell-derived clone

72 hours after infection by lentivirus-expressing sgRNAs with GFP of Boff-p210 Cas9 cell lines, GFP-positive cells were selected by fluorescence-activated cell sorting (FACS) using FACS Aria (BD Biosciences), establishing the cell lines known as Bcr-Abl-EP, Tk-Abl1-EP and Tk-Abl2-EP. Single-cells were seeded in 96-well plate by FACS, establishing four clones with frame-shift mutations that disrupt the *BCR/ABL* sequence. Clone 1 was chosen to establish the cell line known as Bcr-Abl-SC.

### Off-target sequence analysis

To investigate off-target cleavage potentially generated by Cas9 in Bcr-Abl-EP and Bcr-Abl-SC cells, the top five off-target sites (obtained from the crispr.mit.edu website) were analyzed by PCR and Sanger sequencing, just before to start the functional and xenograft experiments. The primers used to amplify the top five predicted off-targets regions are described in Table 2. PCR products were purified using a High Pure PCR Product Purification Kit (Roche) and were sequenced using each forward and reverse PCR primers.

### Apoptosis and cell cycle analysis

Apoptosis was measured by flow cytometry with an annexin V-Dy634 apoptosis detection kit (ANXVVKDY, Immunostep) following the manufacturer's instructions. Briefly, 5 × 10^5^ cells were collected and washed twice in PBS, and labeled with annexin V-DY-634 and non-vital dye propidium iodide (PI), allowing the discrimination of living-intact cells (annexin-negative, PI-negative), early apoptotic cells (annexin-positive, PI-negative) and late apoptotic or necrotic cells (annexin-positive, PI-positive). In parallel, cell distribution in the cell cycle phase was also analyzed by measuring DNA content (PI labeling after cell permeabilization).

### Mouse xenograft tumorigenesis

18 four- to five-week-old female CB.17 SCID mice (Charles River, Barcelona, Spain) were used (six mice per group). Tumor xenografts were induced by subcutaneous injection of cell suspensions containing 8 × 10^6^ cells in 0.2 ml of cellular medium into the mouse flank.

Cells were counted using a Neubauer chamber (VWR) and cellular viability monitored by trypan blue staining (Sigma). This study followed the Spanish and European Union guidelines for animal experimentation (RD 1201/05, RD 53/2013 and 86/609/CEE, respectively). The study received prior approval from the Bioethics Committee of our institution.

In the first mouse group, Boff-p210 Cas9 mock sgRNA cells were injected in the left flank and Bcr-Abl-EP cells in the right flank; in the second group, Boff-p210Cas9 mock sgRNA cells were injected in the left flank and Bcr-Abl-SC cells in the right flank; and, finally, in the third group (controls), Boff-p210 cells were injected in the left flank and Baf/3 in the right flank. Tumor diameters were measured every 2–3 days with a caliper. Tumor volume was calculated as described elsewhere [58] by the formula a^2^bπ/6 (a and b being, respectively, the smallest and the biggest diameters). Mice were sacrificed by anesthesia overdose 24 days after cell injection, upon which the tumors were collected and weighted.

### Statistical analysis

Statistical analysis was performed using GraphPad Prism 6 Software (GraphPad Software). Differences in annexin V labeling were tested using one-way ANOVA and Tukey's multiple comparisons test. Differences in tumor volume over time were tested by two-way ANOVA, and Tukey's multiple comparisons test. Differences in median tumor masses were tested by the Kruskal-Wallis and Mann-Whitney *U* tests, and Dunn's multiple comparisons test. Statistical significance at values of *p* < 0.05 (**) and p < 0.001 (***) was noted.

## SUPPLEMENTARY MATERIALS FIGURES AND TABLE





## Abbreviations

CML: Chronic myeloid leukemia
CRISPR: Clustered regularly interspaced short palindromic repeats
sgRNA: Single-guide RNA
EP: Edited pool
SC: Single edited cell-derived
