# Supplementary material for: The CRISPR/Cas9 system efficiently reverts the tumorigenic ability of BCR/ABL in vitro and in a xenograft model of chronic myeloid leukemia
Source: Oncotarget. 2017 Feb 9;8(16):26027–40. doi: 10.18632/oncotarget.15215 (PMC5432235; doi:10.18632/oncotarget.15215)
Supplement: Supplementary file 1 [file oncotarget-08-26027-s001.pdf]

The CRISPR/Cas9 system efficiently reverts the tumorigenic ability of *BCR/ABL in vitro* and in a xenograft model of chronic myeloid leukemia

Supplementary Materials

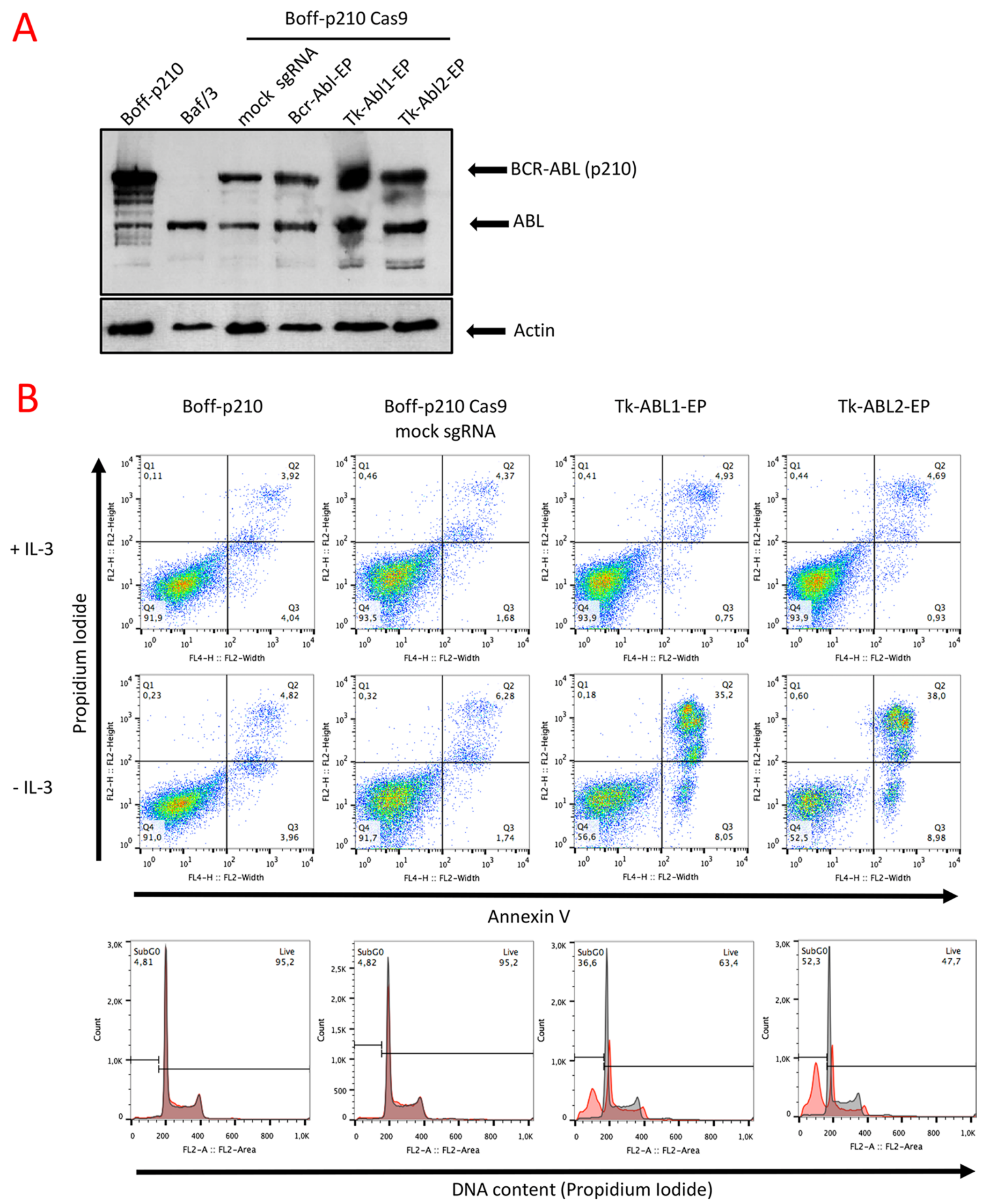

Supplementary Figure 1: Functional analysis of Boff-p210-edited cells with TK-Abl sgRNAs. (**A**) Western blot analysis of BCR-ABL protein expression in Tk-Abl1-EP and Tk-Abl2-EP cells. We observed no changes in BCR-ABL fusion protein expression in any edited cell pool. (**B**) Annexin V/propidium iodide labeling of Boff-p210 cells after four days culture in the presence or absence of IL-3. No edited or mock sgRNA-expressing cells showed IL-3 independent growth. Tk-Abl1-EP and Tk-Abl2-EP cells showed greater annexing V labeling after IL-3 withdrawal. Likewise, larger subG0 populations were observed in the absence of IL-3. No changes were observed in the cell cycles of the Boff-p210 or mock cells, grown with or without IL-3.

A Single-Cell Clones	Sequence	Effect
Wild Type	CACTGGATTTAAGCAGAGTTCAAAAGCCCTTCAGCGGCCAGTAGCATCTGACTTTGAG	
Clone 1 Δ(8) CCTTCAGC/--------	CACTGGATTTAAGCAGAGTTCAAAAGC********GGCCAGTAGCATCTGACTTTGAG	STOP
Clone 2 Δ (4) CAGC/----	CACTGGATTTAAGCAGAGTTCAAAAGCC****AGCGGCCAGTAGCATCTGACTTTGAG	STOP
Clone 3 Ins AGTAG/ Δ CTT	CACTGGATTTAAGCAGAGTTCAAAAGCCagtagCAGCGGCCAGTAGCATCTGACTTTGAG	STOP
Clone 4 Δ (11)	CACTGGATTTAAGCAGAGTTCAAAAGCC ***********AGTAGCATCTGACTTTGAG	STOP

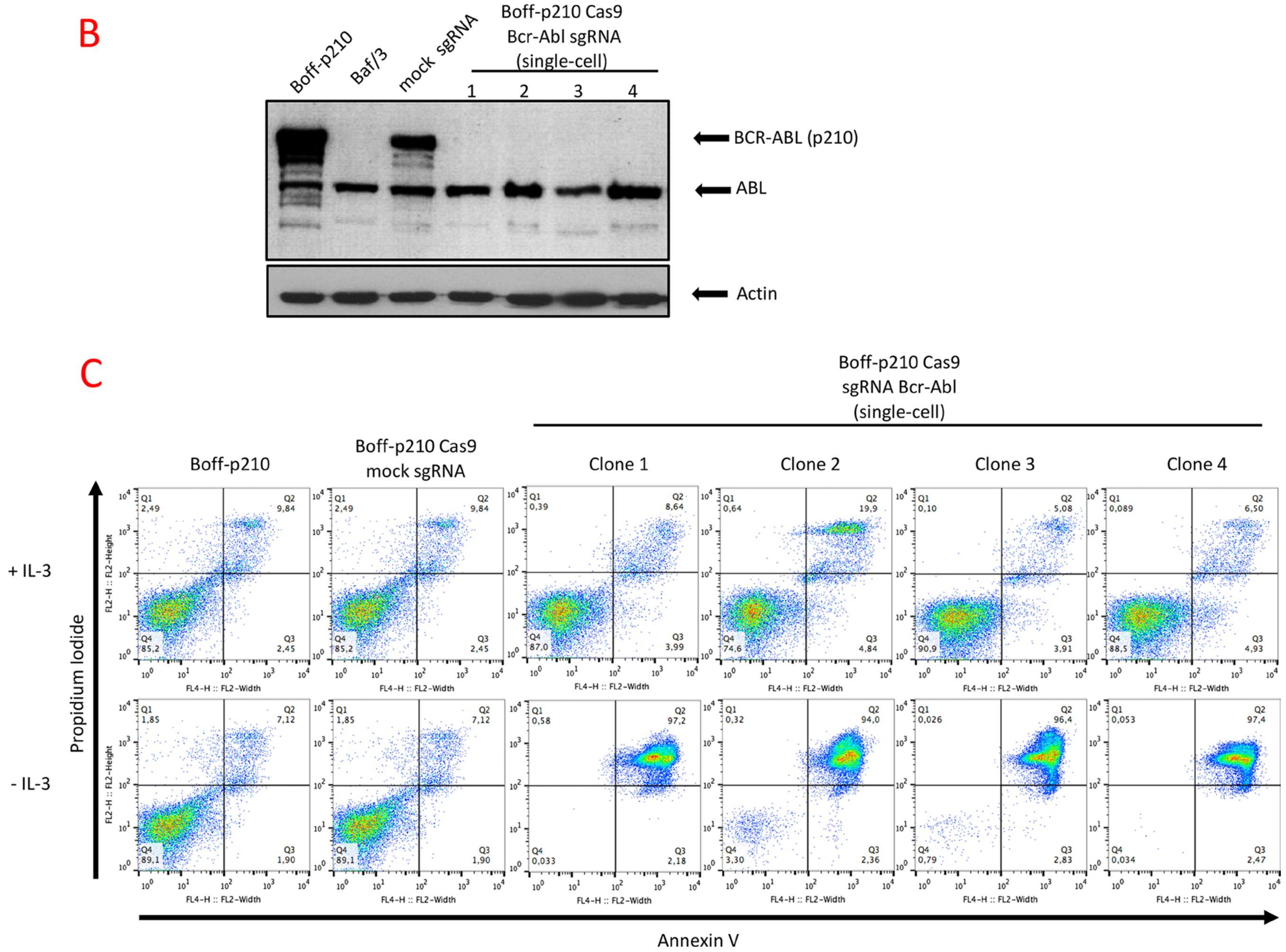

Supplementary Figure 2: Boff-p210 Bcr-Abl sgRNA single edited cell-derived clone analysis and selection. (**A**) Four clones (4/41) carrying mutations generating a premature stop-codon near the Cas9 cleavage point, leading to the premature end of BCR/ABL translation. (**B**) Western blot analysis of BCR-ABL expression of selected clones. In silico-predicted premature end translation resulted in the absence of BCR-ABL expression in the four selected clones. (**C**) Functional analysis of Boff-p210 Bcr-Abl sgRNA single edited cell-derived clones. Four clones with truncated BCR-ABL expression showed a substantial (> 90%) increase in annexin V/propidum iodide labeling in the absence of IL-3. Clone 1 was used to establish the Bcr-Abl-SC cell line.

Supplementary Table 1: Analysis of BCR/ABL sequence from 41 single edited cell-derived clones.
See Supplementary_Table_1
