# Supplementary material for: The CRISPR/Cas9 system efficiently reverts the tumorigenic ability of BCR/ABL in vitro and in a xenograft model of chronic myeloid leukemia
Source: Oncotarget. 2017 Feb 9;8(16):26027–40. doi: 10.18632/oncotarget.15215 (PMC5432235; doi:10.18632/oncotarget.15215)
Supplement: Supplementary file 2 [file oncotarget-08-26027-s002.docx]

Supplementary Table 1: Analysis of BCR/ABL sequence from 41 single edited cell-derived clones.

| **Single-cell Clone**  **Variation** | **Sequence** | **Effect** |
| --- | --- | --- |
| Wild Type | CACTGGATTTAAGCAGAGTTCAAAAGCCCTTCAG**CGG**CCAGTAGCATCTGACTTTGAG |  |
| Clone 1 Ins 5bp / Del 3bp | CACTGGATTTAAGCAGAGTTCAAAAGCCagtag***CGGCCAGTAGCATCTGACTTTGAG | STOP |
| Clone 2 Del 4 | CACTGGATTTAAGCAGAGTTCAAAAGCC****AGCGGCCAGTAGCATCTGACTTTGAG | STOP |
| Clone 3 Del 8bp | CACTGGATTTAAGCAGAGTTCAAAAGC********GGCCAGTAGCATCTGACTTTGAG | STOP |
| Clone 4 Del 11bp | CACTGGATTTAAGCAGAGTTCAAAAGCC ***********AGTAGCATCTGACTTTGAG | STOP |
| Clon 5 WT | CACTGGATTTAAGCAGAGTTCAAAAGCCCTTCAGCGGCCAGTAGCATCTGACTTTGAG | none |
| Clon 6 Ins 5 bp/ Del 3bp | CACTGGATTTAAGCAGAGTTCAAAAGCCagtagCAGCGGCCAGTAGCATCTGACTTTGAG | STOP |
| Clon 7 Ins 665 bp |  | STOP |
| clon 8 Ins 3 bp | CACTGGATTTAAGCAGAGTTCAAAAGCCCTTCttcAGCGGCCAGTAGCATCTGACTTTGAG | add 1 aa |
| Clon 9 WT | CACTGGATTTAAGCAGAGTTCAAAAGCCCTTCAGCGGCCAGTAGCATCTGACTTTGAG | none |
| Clon 10 WT | CACTGGATTTAAGCAGAGTTCAAAAGCCCTTCAGCGGCCAGTAGCATCTGACTTTGAG | none |
| Clon 11 Del 18 bp | CACTGGATTTAAGCAGAGTTCA******************GTAGCATCTGACTTTGAG | remove 6 aa |
| Clon 12 Del 11 bp | CACTGGATTTAAGCAGAGTTCAAAAGCC ***********AGTAGCATCTGACTTTGAG | STOP |
| Clon 13 Ins 5 bp / Del 3bp | CACTGGATTTAAGCAGAGTTCAAAAGCCagtag***CGGCCAGTAGCATCTGACTTTGAG | STOP |
| Clon 14 Del 1bp | CACTGGATTTAAGCAGAGTTCAAAAGCC*TTCAGCGGCCAGTAGCATCTGACTTTGAG | STOP |
| Clon 15 A/G | CACTGGATTTAAGCAGAGTTCAAAAGCCCTTCAaCGGCCAGTAGCATCTGACTTTGAG | none |
| Clone 16 WT | CACTGGATTTAAGCAGAGTTCAAAAGCCCTTCAGCGGCCAGTAGCATCTGACTTTGAG | none |
| clone 17 Del C | CACTGGATTTAAGCAGAGTTCAAAAGCCCTT*AGCGGCCAGTAGCATCTGACTTTGAG | STOP |
| Clon 18 Del 8bp | CACTGGATTTAAGCAGAGTTCAAAAGC********GGCCAGTAGCATCTGACTTTGAG | STOP |
| Clon 19 Del 11bp | CACTGGATTTAAGCAGAGTTCAAAAGCC***********AGTAGCATCTGACTTTGAG | STOP |
| Clone 20 Del 8bp | CACTGGATTTAAGCAGAGTTCAAAAGC********GGCCAGTAGCATCTGACTTTGAG | STOP |
| Clone 21 Del T | CACTGGATTTAAGCAGAGTTCAAAAGCCCT*CAGCGGCCAGTAGCATCTGACTTTGAG | STOP |
| clon 22 Del 11bp | CACTGGATTTAAGCAGAGTTCA***********GCGGCCAGTAGCATCTGACTTTGAG | STOP |
| Clon 23 Del 11bp | CACTGGATTTAAGCAGAGTTCAAAAGCC***********AGTAGCATCTGACTTTGAG | STOP |
| Clone 24 WT | CACTGGATTTAAGCAGAGTTCAAAAGCCCTTCAGCGGCCAGTAGCATCTGACTTTGAG | none |
| Clone 25 WT | CACTGGATTTAAGCAGAGTTCAAAAGCCCTTCAGCGGCCAGTAGCATCTGACTTTGAG | none |
| Clon 26 Del 18 bp | CACTGGATTTAAGCAGAGTTCA******************GTAGCATCTGACTTTGAG | remove 6 aa |
| Clone 27 Ins 1bp | CACTGGATTTAAGCAGAGTTCAAAAGCCCTTtCAGCGGCCAGTAGCATCTGACTTTGAG | STOP |
| Clone 28 WT | CACTGGATTTAAGCAGAGTTCAAAAGCCCTTCAGCGGCCAGTAGCATCTGACTTTGAG | none |
| Clon 29 Del 8bp | CACTGGATTTAAGCAGAGTTCAAAAGC********GGCCAGTAGCATCTGACTTTGAG | STOP |
| Clon 30 Del 8bp | CACTGGATTTAAGCAGAGTTCAAAAGC********GGCCAGTAGCATCTGACTTTGAG | STOP |
| Clone 31 WT | CACTGGATTTAAGCAGAGTTCAAAAGCCCTTCAGCGGCCAGTAGCATCTGACTTTGAG | none |
| Clone 32 WT | CACTGGATTTAAGCAGAGTTCAAAAGCCCTTCAGCGGCCAGTAGCATCTGACTTTGAG | none |
| Clone 33 Del 4bp | CACTGGATTTAAGCAGAGTTCAAAAGCC****AGCGGCCAGTAGCATCTGACTTTGAG | STOP |
| Clone 34 Del 3bp | CACTGGATTTAAGCAGAGTTCAAAAGCCC***AGCGGCCAGTAGCATCTGACTTTGAG | remove 1 aa |
| Clone 35 Ins >500 bp |  | Stop |
| Clone 36 Del 8 bp | CACTGGATTTAAGCAGAGTTCAAAAGC********GGCCAGTAGCATCTGACTTTGAG | STOP |
| Clone 37 Del 4 bp | CACTGGATTTAAGCAGAGTTCAAAAGCC****AGCGGCCAGTAGCATCTGACTTTGAG | STOP |
| Clone 38 Del 1bp | CACTGGATTTAAGCAGAGTTCAAAAGCCCT*CAGCGGCCAGTAGCATCTGACTTTGAG | STOP |
| Clone 39 Del 11bp | CACTGGATTTAAGCAGAGTTCAAAAGCC ***********AGTAGCATCTGACTTTGAG | STOP |
| Clone 40 Ins 5bp / Del 3 bp | CACTGGATTTAAGCAGAGTTCAAAAGCCagtag***CGGCCAGTAGCATCTGACTTTGAG | STOP |
| Clon 41 Del 7 bp | CACTGGATTTAAGCAGAGTTCAAA*******CAGCGGCCAGTAGCATCTGACTTTGAG | STOP |
